# The Risk of a Mosquito-Borne Infectionin a Heterogeneous Environment

**DOI:** 10.1371/journal.pbio.0020368

**Published:** 2004-10-26

**Authors:** David L Smith, Jonathan Dushoff, F. Ellis McKenzie

**Affiliations:** **1**Fogarty International Center, National Institutes of HealthBethesda, MarylandUnited States of America; **2**Ecology and Evolutionary Biology, Princeton UniversityPrinceton, New JerseyUnited States of America

## Abstract

A common assumption about malaria, dengue, and other mosquito-borne infections is that the two main components of the risk of human infection—the rate at which people are bitten (human biting rate) and the proportion of mosquitoes that are infectious—are positively correlated. In fact, these two risk factors are generated by different processes and may be negatively correlated across space and time in heterogeneous environments. Uneven distribution of blood-meal hosts and larval habitat creates a spatial mosaic of demograPhic sources and sinks. Moreover, mosquito populations fluctuate temporally, forced by environmental variables such as rainfall, temperature, and humidity. These sources of spatial and temporal heterogeneity in the distribution of mosquito populations generate variability in the human biting rate, in the proportion of mosquitoes that are infectious, and in the risk of human infection. To understand how heterogeneity affects the epidemiology of mosquito-borne infections, we developed a set of simple models that incorporate heterogeneity in a stepwise fashion. These models predict that the human biting rate is highest shortly after the mosquito densities peak, near breeding sites where adult mosquitoes emerge, and around the edges of areas where humans are aggregated. In contrast, the proportion of mosquitoes that are infectious reflects the age structure of mosquito populations; it peaks where old mosquitoes are found, far from mosquito breeding habitat, and when mosquito population density is declining. Finally, we show that estimates for the average risk of infection that are based on the average entomological inoculation rate are strongly biased in heterogeneous environments.

## Abstract 2

A modeling approach reveals that incorporating the demography and behavior of mosquitoes can substantially change estimates of the risk of infection from diseases such as malaria

## Introduction

Understanding the spatiotemporal distribution of risk for mosquito-borne infections is an important step in planning and implementing effective infection control measures (Greenwood 1989; Charlwood et al. 1998; Chadee and Kitron 1999; Focks et al. 1999; Mendis et al. 2000; Carter 2002; Killeen et al. 2003). Remote sensing, geographical information systems, and predictive algorithms have made it possible to develop coarse-grained maps of vector habitat (Pope et al. 1994; Beck et al. 1997; Kitron 1998; Rogers et al. 2002), and epidemiological studies have identified statistical risk factors for human infection or disease (Snow et al. 1998; Ghebreyesus et al. 2000; Snow and Gilles 2002). Mathematical models can bridge the gaps between landscape ecology, vector biology, and human epidemiology, linking large-scale maps to individual risk in local human populations at spatial scales ranging from 10 m up to 10 km. At these spatial scales, transmission dynamics for vector-borne infections are linked to the seasonal dynamics, demography, and behavior of adult female mosquitoes, as well as the spatial distribution of larval habitat and blood hosts (Bidlingmyer 1985). Given a map of potential or actual mosquito sources and human habitation, what factors determine where and when the risk of a mosquito-borne infection is highest?

The risk of a mosquito-borne infection is estimated by the entomological inoculation rate (EIR): the number of bites by infectious mosquitoes per person per day (Macdonald 1957). EIR is the product of the human biting rate (HBR)—the number of bites by vector mosquitoes per person per day—and the proportion of mosquitoes that are infectious (PIM) (e.g., for malaria transmission, the sporozoite rate) (Birley and Charlwood 1987). We focus on the processes that generate patterns in the two components of EIR in temporally and spatially heterogeneous mosquito populations. We show that HBR and PIM peak at different times and places. We also show that estimates of average EIR in variable environments generate biased estimates of the relationship between EIR and the proportion of humans that are infected (Dye and Hasibeder 1986).

We develop theory to illustrate simple patterns in the EIR in heterogeneous environments, focusing on EIR's separate components. We follow the a priori approach of Ross, developing simple mathematical models as tools for qualitative and quantitative reasoning (McKenzie 2000). We develop a basis for micro-epidemiological models for mosquito-borne infections that can be combined with surveys of larval habitat to map the local risk of mosquito-borne infections (Greenwood 1989). Effective use and refinement of such maps depend on an understanding of the dynamics and behavior of specific mosquito populations and the transmission of specific infectious agents (Focks et al. 1999).

## Results

### Temporal Heterogeneity

**Figure 1:**
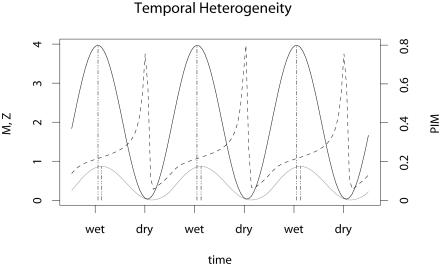
Dynamics with Temporal Heterogeneity The components of EIR follow different trends when mosquito populations vary temporally. Mosquito density (solid black) forms the dominant component of HBR. The density of infected mosquitoes (solid gray) peaks shortly after the density of mosquitoes (dotted vertical lines align the peaks). In contrast, the proportion of infectious mosquitoes (dashed) peaks while the mosquito population is declining. Seasonal mosquito emergence was modeled to have an long-term average *M/H* ≈ 2 (*K* = 2 and *H* = 1). The ticks on the x-axis mark the peaks of the wet and dry seasons.

Fluctuating mosquito density affects EIR through changes in HBR: transmission increases as mosquito density increases. Following an increase in the rate at which adult mosquitoes emerge, mosquito density and HBR peak (illustrated in Figure 1). The peak in EIR and the density of infected mosquitoes follow the peak in total mosquito density because it takes time for an infectious agent to spread through the human and mosquito populations. Increased HBR leads to secondary increases in the proportion of infected humans, and thus to increases in PIM. As the density of infected mosquitoes declines, decreasing transmission is followed by a decline in the prevalence of infection in humans.

In contrast, larger fluctuations in PIM are generated by the shifting age distribution in fluctuating mosquito populations. Adults emerge uninfected, but they become infected some time after biting infectious humans. Growing populations are dominated by young, uninfected mosquitoes, while shrinking populations are dominated by older mosquitoes. Since the proportion of mosquitoes that are infected and infectious increases with the age of the mosquito, PIM is a proxy for the age distribution of mosquito populations. As populations decline, surviving mosquitoes continue to bite and oviposit but few young mosquitoes emerge, so declining populations have a larger fraction of old mosquitoes. Thus, PIM increases during the dry season as mosquito populations, HBR, and EIR decline.

### Spatial Heterogeneity

The distribution of adults is determined by the distribution of larval habitat, the distribution of blood hosts, and the alternating activities of blood-meal-seeking and oviposition. When mosquito emergence rates and human population distributions are constant over time, the distribution of mosquitoes reaches a static spatial distribution. We focus on the patterns that form along a transect.

**Figure 2:**
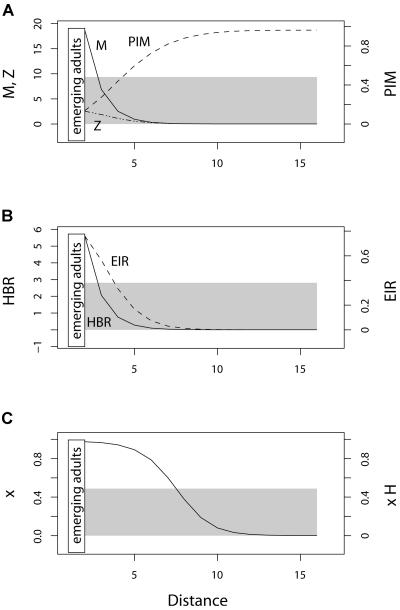
Statics with Homogeneous Humans and Heterogeneous Mosquitoes The components of EIR follow different trends when larval habitat is distributed at a single point and humans are uniformly distributed (the gray background illustrates the human distribution). (A) Mosquito density (solid) declines monotonically, but PIM (dashed) increases monotonically. The density of infected mosquitoes (*Z,* dotted) also declines monotonically. (B) HBR (solid) and EIR (dashed) both decline monotonically away from the source, reflecting the steep gradient in mosquito density. (C) The density of infected humans (dashed) and prevalence of infection in humans (solid) also decline monotonically (the curves coincide).

**Figure 3:**
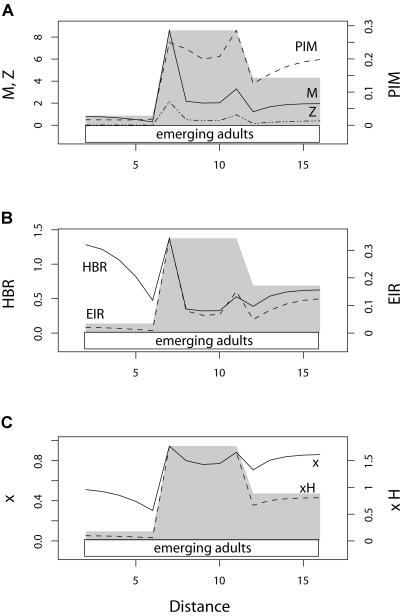
Statics with Heterogeneous Humans and Homogeneous Mosquitoes HBR and EIR reflect mosquito movement and human distribution patterns when larval habitat is evenly distributed but humans have a low–high–medium distribution, such as a town with rural and suburban populations on either side (the gray background illustrates the human distribution). (A) Mosquito density (solid) is highest in town, peaks at the edges of town, and dips just outside of town. PIM (dashed) and the density of infected mosquitoes (*Z,* dotted) follow similar patterns. (B) HBR (solid) and EIR (dashed) are both high on the low-density side of town and lowest on the medium-density side of town, with peaks just inside town and troughs just outside of town. (C) The density of infected humans (dashed) and the prevalence of infection in humans (solid) peak at the edge of town, but prevalence of infection in humans is less variable than HBR or EIR.

**Figure 4:**
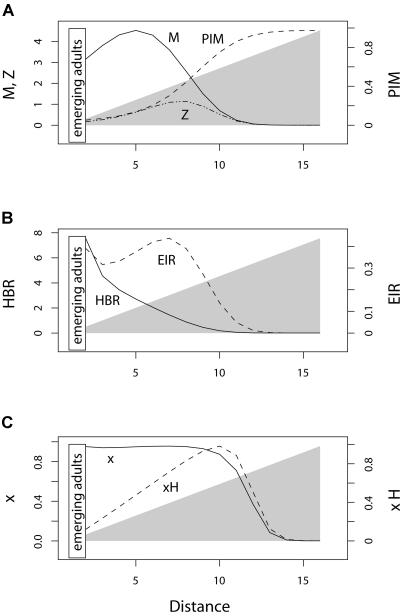
Statics with Heterogeneous Humans and Mosquitoes When human density increases smoothly away from a larval habitat (the gray background illustrates the human distribution), the patterns of EIR components reflect heterogeneity in the distribution of larval habitat and human populations. (A) Mosquito density (solid) peaks an intermediate distance away from the source. The peak density of infected mosquitoes (*Z,* dotted) is further from the source because PIM (dashed) increases monotonically away from the source. (B) HBR (solid) decreases monotonically away from the source, reflecting mosquito density, but EIR (dashed) has a minor peak away from the source. (C) The density of infected humans (dashed) peaks away from the source, but the prevalence of infection in humans (solid) remains relatively constant near the source, dropping off sharply further away.

In Figure 2, we assume a single point source for mosquitoes and a homogeneous distribution of humans. In Figure 3, the same number of adult mosquitoes emerges, but the spatial distribution of larval emergence is uniform along the transect and the distribution of humans varies: human density is low at one end, high at intermediate locations, and intermediate at the opposite end, approximating a small town with fewer dwellings on the edge nearest a swampy area. In Figure 4 we combine the two kinds of spatial heterogeneity.

### Gradients in EIR Away from Larval Habitat

When mosquitoes emerge from a point source, the density of mosquitoes tends to decline with distance from larval habitat, such as a gradient along a transect away from a swamp or river (Figure 2A). The shape of the gradient is determined by the emergence rate of adult mosquitoes, the mortality of existing mosquitoes, and random drift away from the source. In contrast, PIM increases monotonically away from the source because of a shift in the age distribution and parity of mosquitoes (Figure 2B). Young mosquitoes tend to be close to their birthplace because they have moved less; older mosquitoes have moved more and so are dispersed further from the source, on average. The spatial distribution of HBR and EIR reflect the gradients in mosquito density, not the gradient in PIM (Figure 2A and 2B). The prevalence of infection in humans declines monotonically with distance from the mosquito source (Figure 2C).

### Heterogeneous Distributions of Humans

When human populations are distributed heterogeneously, but the larval habitat of mosquitoes is distributed uniformly, adult mosquito distributions become heterogeneous because mosquitoes tend to aggregate around humans. Whether this leads to an increase in HBR depends on whether mosquito distributions become more aggregated than the distribution of their human hosts. HBR tends to increase when searching mosquitoes move rapidly through sparse human populations and linger in areas with dense human populations. Thus, mosquito distributions tend to become more aggregated than human distributions when the mosquito species is long-lived with long daily flight distances (see below).

We illustrate this principle for one particular set of parameters that leads to increased mosquito aggregation. The human population is distributed heterogeneously in blocks of low, high, and medium density, approximating a town with a rural population on one side and an intermediate-density population on the other. The distribution of adult mosquitoes is influenced by the distribution of humans (Figure 3A). Aggregations of mosquitoes form spontaneously at the edges of human settlements simply because mosquitoes tend to move until they find a host. We note that the major peaks in HBR are away from town, where human population density is lowest, and at the edge of town, where human population density is highest (Figure 3B). EIR also peaks at the edge of town, but it is lowest on the low-human-density side of town. With these movement rules, the mosquitoes found on the side of town with low human density tend to be younger, hence PIM is low (Figure 3A). The prevalence of infection in humans is lowest overall in the patches with low human density (Figure 3C). This model also makes the surprising prediction that the risk of infection is lowest just outside the edge of town: the sharp difference in human density at the edge leads to a strong tendency for mosquitoes to be drawn into, rather than away from, town, decreasing HBR and PIM (Figure 3B and 3C).

### Heterogeneous Larval Habitat and Human Population

When mosquitoes and humans are distributed unevenly, the distribution of mosquitoes and risk may be dominated either by proximity to larval habitats and gradients away from them or by the tendency of mosquitoes to aggregate around humans. The realized pattern depends on the relative distribution of larval habitat and humans, and whether mosquito aggregation around humans increases HBR. We illustrate one kind of pattern for parameters that lead to increased HBR. In this case, human density increases away from larval habitat. The density of mosquitoes peaks a short distance from the source, and the density of infected mosquitoes peaks slightly further away (Figure 4A). HBR declines monotonically away from the source, but EIR peaks at an intermediate distance (Figure 4B). The density of infected humans peaks well away from the source, but the fraction of infected humans remains relatively constant near the source, declining abruptly at distances beyond the peak in infected humans (Figure 4C). Despite the sharp peaks in risk, PIM displays a robust monotonic increase with distance away from the source (Figure 4A). If the gradient is reversed, so that human density decreases with the distance away from larval habitat, mosquitoes remain close to the source and mosquito aggregation is exaggerated, compared with Figure 2 (data not shown).

### Measuring EIR in Heterogeneous Environments

**Figure 5:**
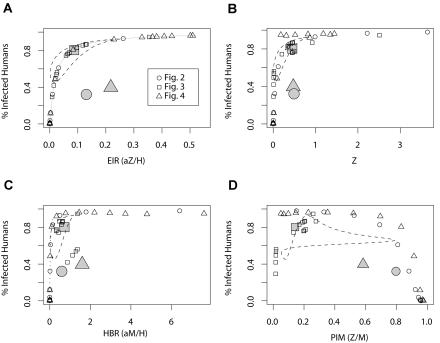
The Relationship between EIR and Human Prevalence, with Heterogeneity (A) EIR and the prevalence of infection in humans have a tidy relationship among patches; each small symbol is from a single patch in Figures 2–4. The relationship, given by equation 1, is plotted in gray. The phase plane of the dynamic relationship over time from Figure 1 is plotted with dashed lines. Average EIR is plotted against the average prevalence (large symbols). Predicting the average prevalence of human infection from average EIR leads to underestimates. (B–D) The density of infectious mosquitoes *(Z)* (B), HBR (HBR = *aM*/*H*) (C), and PIM (PIM = *Z*/*M*) (D) are plotted against the proportion of humans who are infected and infectious. PIM is a particularly bad measure of the risk of infection; in heterogeneous habitats, it peaks far from larval habitat, where mosquito density and prevalence of infection in humans is lowest. This accounts for the large number of points where PIM is high, but the proportion of infectious humans is low.



Variability in EIR across a landscape can lead to systematic bias in the estimation of risk. In Figure 5, we plot local EIR and its components against the local prevalence of infection in humans for the individual patches in Figures 2–4. We also plot the average EIR for each transect. In addition, we overlay the temporal patterns from Figure 1 as a phase diagram. We note that local EIR and local prevalence of infection in humans at equilibrium, x¯, have a clear nonlinear relationship given by the following formula:

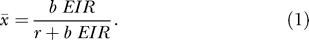



In contrast, the relationship between average EIR and average prevalence of infection in humans is biased, such that average prevalence always falls below the true relationship (Figure 5A). The bias is due to an inherent mathematical property of nonlinear relationships known as Jensen's inequality (Krantz 1999). Since the relationship between EIR and the prevalence of infection in humans is concave down, aggregating estimates of EIR in variable habitat will always underestimate the true relationship, sometimes spectacularly (Ruel and Ayres 1999).

The local density of infectious mosquitoes (Figure 5B) and the local HBR (Figure 5C) provide reasonably good estimates of risk. In both cases, spatial heterogeneity in human density or PIM is a substantial source of variability in measures of average risk. In contrast, PIM displays no clear pattern along the transect (Figure 5C). The patches in which PIM is high but the prevalence of infection in humans is low are all far from larval habitat.

### Sensitivity Analysis

The patterns illustrated in Figures 2–4 are based on a single set of entomological parameters in order to facilitate comparisons among situations in which only the distributions of mosquitoes and hosts vary. The distribution of risk will change for different values of the parameters. We explored the effects of mosquito movement and the duration of the incubation period on the distribution of risk (below and Protocol S1).

The tendency of mosquitoes to aggregate at the edges of a town or away from larval habitat depends on mosquito searching behavior and demography. Three important parameters that affect these patterns are the maximum daily flight distance of a mosquito, mosquito longevity, and mosquito searching efficiency. The distribution of a mosquito cohort initially reflects the distribution of larval habitat. As mosquitoes search for hosts, the distribution of the cohort shifts to reflect the distribution of human hosts. These tendencies are also reflected in the static spatial distributions of mosquitoes. The distribution of long-lived mosquitoes with long daily flight distances will tend to reflect the underlying distribution of humans, while the distribution of short-lived mosquitoes with short flight distances will tend to reflect the distribution of larval habitat. Mosquito searching efficiency determines the relative rates of movement through habitats that vary in human density. A strong tendency for mosquitoes to aggregate at the edges of dense human populations occurs when mosquitoes move quickly through areas that are sparsely populated by humans and linger in areas that are heavily populated. In other words, mosquitoes tend to become more aggregated than their hosts, increasing HBR, when mosquito searching is relatively inefficient at low human densities.

The distribution of relative risk also changes with the time required for incubation of the infectious agent, with mechanically transmitted agents at one extreme. When all else is equal, HBR is higher in areas in which human density is low, since human population density is in the denominator of HBR. On the other hand, HBR may decline in low-human-density areas because mosquitoes tend to move up a gradient of human population density in search of a blood-meal host. Such migration will tend to lower the average age of mosquitoes in low-human-density patches, especially near the edge of a town. This will tend to lower PIM for infectious agents with a long incubation period. In contrast, PIM for mechanically transmitted infectious agents will not be as strongly affected, so in comparison, the relative risk may be higher at that same edge of town.

## Discussion

EIR is generally considered to be the best estimate of the risk of mosquito-borne infections, but EIR varies over space and time. EIR varies spatially because larval habitat and blood-meal hosts are heterogeneously distributed across a landscape. Temporal variability is generally driven by weather, especially rainfall, temperature, and humidity. To compound the problem, heterogeneity in human feeding over short distances can be caused by vector preferences for individual humans based on odor or other cues (Takken and Knols 1999; Kelly 2001). Heterogeneous biting has important implications for the dynamics and control of mosquito-borne infections (Dietz 1980; Dye and Hasibeder 1986; Woolhouse et al. 1997).

Heterogeneous biting also has important implications for the measurement of EIR. Estimates of EIR may vary substantially over short distances depending on the place and time at which the measurement is made. Depending on the method used, EIR may also vary with the relative attractiveness of the human bait. Our mathematical models have shown that average EIR in heterogeneous environments gives a strongly biased estimate of average risk, even when local estimates of EIR provide a perfect measure of local risk. The bias is unavoidable because the relationship between EIR and the proportion of humans who are infected is nonlinear, which leads to a bias due to Jensen's inequality (Krantz 1999). A similar bias is likely to arise when estimating risk for other infectious diseases, a problem that is pervasive and generally underappreciated in epidemiology and public health (Ruel and Ayres 1999). Therefore, mathematical models are an indispensable tool for the design and interpretation of field studies (Becker 1989).

Mathematical models provide a sound approach to understanding risk and planning for control in heterogeneous environments, especially when the models are based on the ecology of the local vector populations and a sound understanding of the entomological parameters relevant for transmission (Killeen et al. 2000a, 2000b). Creating micro-epidemiological maps for the distribution of risk would involve mapping larval habitat and humans, and combining these maps with an understanding of the temporal dynamics, blood-meal-seeking behavior, and oviposition habits of mosquitoes. A critical assumption of the models described here is that mosquitoes are able to oviposit everywhere. Vector species may be very selective about where they oviposit, forcing a return to larval habitat to oviposit between successive bites. Thus, the heterogeneous distribution of oviposition sites may also affect the distribution of risk.

A dominant component of EIR is the density of mosquito vectors relative to human density. Our models show that mosquito densities and the proportion of humans who carry a mosquito-borne infection decline with distance away from larval habitat because of random movement and mosquito mortality. Such patterns have been documented by numerous field studies (Trape et al. 1992; Hii et al. 1997; Charlwood et al. 1998; Clarke et al. 2002; Minakawa et al. 2002; Keating et al. 2003; Staedke et al. 2003; Konradsen et al. 2003; van der Hoek et al. 2003). The steepness of the gradient in EIR varies, depending on the ecology of the vector (Hii et al. 1997).

Unlike the patterns in human biting, the proportion of mosquitoes that are infectious depends on the age structure of the mosquito population. The likelihood of infection in mosquitoes shows a strong association with the age or parity of mosquitoes (Lines et al. 1991). The average age differs in growing, stable, and declining populations (Aron and May 1982). Our models also predict that the proportion of infectious mosquitoes increases monotonically with the distance away from sources of emerging adults; young, pre-gravid mosquitoes are found more frequently near larval habitat, while older mosquitoes are found further away. Such patterns have also been observed in the field (Charlwood et al. 1998).

Mosquito aggregation around dense human populations depends on the details of mosquito searching behavior. Long-lived mosquitoes with long flight distances tend to become more aggregated than their human hosts over intermediate distances. For example, EIR may peak at the edges of a village, as has been documented by one field study (Ribeiro et al. 1996). At larger spatial scales, increasing human density may decrease EIR; one field study concluded that human density was protective against disease (Snow et al. 1998). We have emphasized heterogeneous biting that arises from proximity to larval habitat and from mosquito aggregation due to blood-meal-seeking behavior. In our models, aggregation is generated by the tendency of mosquitoes to migrate more slowly when blood-meal hosts are readily available. Aggregation in human biting may be enhanced if mosquitoes fly toward humans that are more attractive at medium and long distances (Ansell et al. 2002; Mukabana et al. 2002). It remains to be seen how these factors interact; for instance, at what distances are preferred hosts more attractive to mosquitoes (Ansell et al. 2002)?

The use of remote sensing and GIS provides a potentially powerful tool for understanding the distribution of mosquito-borne infections at large spatial scales, but dynamics and control of mosquitoes and mosquito-borne infections occur locally. These technologies will be most effective if they are coupled with micro-epidemiological models of malaria, dengue, and other mosquito-borne infections (Greenwood 1989). Such models can predict variability in local risk based on the distribution of larval habitat, the distribution of humans, and the demography and behavior of the local vectors. To generate realistic predictions for the distribution of risk, it is necessary to understand where and when adult mosquitoes will emerge, and how blood-meal-seeking and the distribution of humans will affect the distribution of HBR. It follows that a knowledge of mosquito demography and behavior should play a central role in the surveillance and control of mosquito-borne infections.

## Materials and Methods

### 

We use mathematical models strategically, to illustrate general principles that may apply to many mosquito-borne infections, not to make predictions about the distribution of a particular infectious agent or the incidence of disease. The models we present and analyze are based on the models for malaria infection developed by Ross (1911). We generate a suite of complex models by elaboration, adding a realistic incubation period, temporal heterogeneity, mosquito movement, patchy space, and spatial heterogeneity (Black and Singer 1987). By comparing models, we associate an effect with a factor. First, we allow mosquito birth rates to vary temporally, and focus on the temporal changes in the components of EIR (Aron and May 1982). Next, we illustrate how spatial variability in the distribution of larval habitat generates source–sink relationships in landscapes and leads to variability in the spatial distribution of HBR and PIM. Then, we explore the consequences of heterogeneous human distributions. Host-seeking behavior by mosquitoes can produce mosquito distributions that are more (or less) aggregated than the distribution of humans, generating an uneven distribution in risk. Thus, we develop conceptual models to illustrate which components of the vector biology determine the distribution of risk.

#### The model

Let *x* denote the proportion of humans who are infected and infectious and *H* denote the population density of humans. We assume that the human infectious period is exponentially distributed with average duration of infection 1/*r*. Thus, we are following Ross in developing a model for infection ignoring superinfection, immunity, and clinical disease (Fine 1975; Aron and May 1982; Cohen 1988; Dietz 1988).

We extend the Ross model by adding temporal variability in mosquito density. Let *ɛ*(*t*) denote the rate adult female mosquitoes emerge from larval habitat; we do not assume that the emergence of adults is explicitly linked to the density of adult mosquitoes. Let *M* denote the population density of mosquitoes, *Z* the density of infectious mosquitoes, and *z* = *Z*/*M* the proportion of mosquitoes that are infectious. We assume that the mosquito lifespan is exponentially distributed with a mean lifetime of 1/*g* d.

We incorporate a realistic incubation period by subdividing the incubation period into *n* stages of equal duration; the proportion of mosquitoes that are infected and incubating in stage *k* is denoted *y_k_,* and the density of mosquitoes in that stage is *Y_k_*. We assume the incubation period has mean of 1/*q* d. The probability of surviving the incubation period is (*qn*/(*qn* + *g*))*^n^* (approximately *e* ^−*g*/*q*^ for large *n*), and the duration of the incubation period (for surviving mosquitoes) has a Gamma distribution with mean 1/*q* and variance 1/(*q* ^2^ *n*); for the numerical simulations, we use *n* = 64 (Protocol S1). The larger *n* is, the smaller the variance is. In the limit as *n* approaches infinity, the dynamics approach a fixed time delay.

Let *a* denote the human feeding rate, the number of human bites per mosquito per day, *b* denote the probability an uninfected human becomes infected from a single bite from an infectious mosquito, and *c* denote the probability that a mosquito becomes infected from biting an infectious human host.

The transmission dynamics of mosquito-borne infections are complex, and it is easy to lose sight of what terms such as EIR and HBR actually mean. HBR is the number of bites received by a human each day. Thus, it is the product of the human feeding rate, *a*—the number of human blood meals per mosquito per day—and the number of mosquitoes per human (i.e., HBR = *aM*/*H*). Therefore, when mosquito density changes, HBR changes proportionally. In contrast, EIR is the number of infectious bites per human per day. Thus, it is the product of PIM and HBR (i.e., EIR = *z*HBR = *azM*/*H*). Table 1 lists variable and parameter names and other important terms for the models.

**Table 1:**
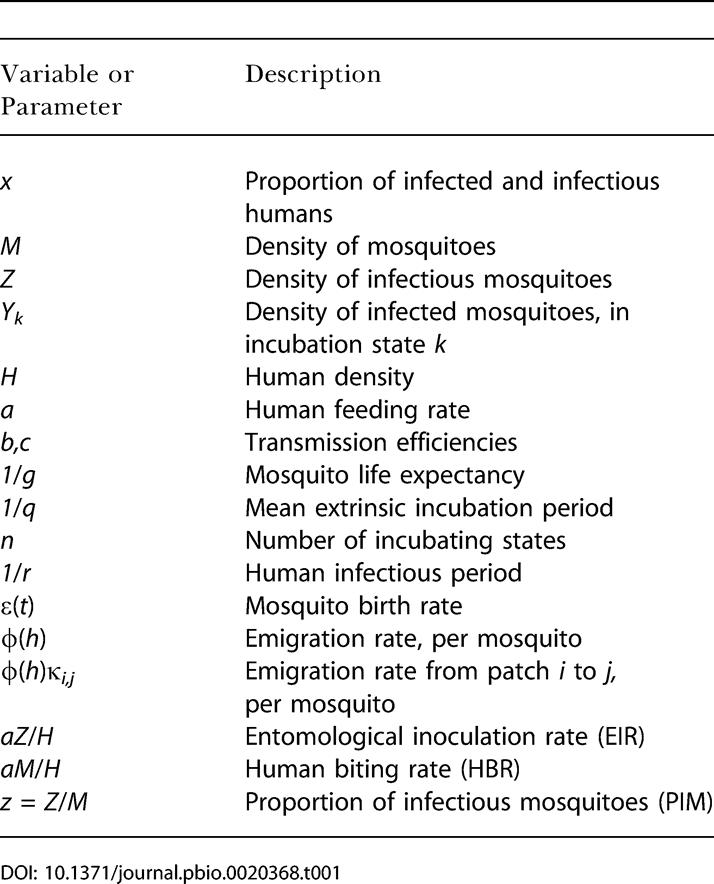
Variables and Parameters Used in the Model

The dynamic process is embedded into a spatial context by subdividing a landscape into a set of patches linked by the movement of mosquitoes. The subscript *i* is added to variable names to denote the value in the *i* ^th^ patch. Thus, *H_i_* denotes local human population density and *x**_i_*** the local prevalence of infection in humans. Similarly, *M_i_* denotes local mosquito population density, and *Z_i_* denotes the density of infectious mosquitoes. The density of infected mosquitoes in patch *i* and incubation stage *k* is denoted *Y_i,k_*. This deterministic approach to incorporating space has some limitations (Mollison 1984, 1986; Durrett and Levin 1994a, 1994b).

Larval habitat and human distributions form a template that determines mosquito distributions and the distribution of risk. The emergence rate of adults in the *i* ^th^ patch is *ɛ_i_*(*t*); the emergence of adult female mosquitoes depends predictably on time and location. Following emergence, female mosquitoes spread into surrounding areas seeking blood hosts; they feed, oviposit, and then repeat the cycle. We assume that heterogeneity in larval habitat takes the form of differences in quality of larval development rather than availability of places to oviposit. In other words, we assume that suitable sites for oviposition are distributed homogeneously throughout the habitat, but that patches may vary in the successful development of adults. Some patches may produce no adults. Heterogeneity in the availability of oviposition habitat would affect the distribution of risk because mosquitoes would alternate between finding a place to oviposit and finding a blood meal. If oviposition were not possible in most patches, those that allowed oviposition would become focal points for mosquito aggregation. Thus, these results apply mainly to mosquito species for which heterogeneous availability of oviposition sites is relatively unimportant for the distribution of risk.

We assume that humans do not move among patches. The density of humans and the productivity of the larval habitat may vary over space. As we change the distribution of humans and larval habitat to explore the effects of spatial heterogeneity, we hold the total emergence rate of adult mosquitoes per human constant; only the distribution of humans and adult mosquito emergence changes.

We assume that mosquitoes are more likely to stay in a patch if they encounter a human, and that they are more likely to find humans where humans are more abundant. Let Φ(*H_i_*) denote the per capita emigration rate of mosquitoes away from patch *i* regardless of infection status. We assume that Φ(*H*) is a decreasing function of *H;* the more humans, the less likely mosquitoes are to leave a patch in search of another blood-meal host. Thus, mosquitoes move more rapidly through patches with low human densities. A parameter, *κ_i,j_,* describes the fraction of mosquitoes leaving patch *i* that fly to patch *j,* and Σ_*j*_κ_*i,j*_ = 1. Thus, the rate that mosquitoes move from patch *i* to patch *j* is Φ(*H_i_*)*κ_i,j_M_i_*.

The transmission dynamics are described by the following set of equations:



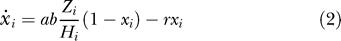





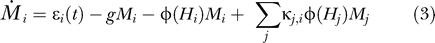





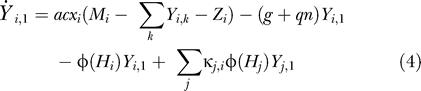





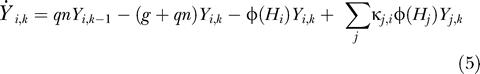





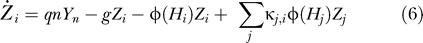



This patch-based modeling framework is suitable for modeling an array or grid of contiguous habitat or an arbitrary network of patches.

#### Numerical solutions

Our intent is to focus on the effects of temporal and spatial heterogeneity. Consequently, we have used a single set of mosquito life-history parameters and a single duration of infection in humans. The parameters are roughly consistent with Anopheles gambiae and the infectious period for malaria (*a* = 0.3; *b* = *c* = 0.5; 1/*g* = 1/*q* = 10 d). The human infectious period for this case is 100 d (*r* = 0.01), roughly consistent with malaria.

Constant mosquito populations were modeled using a constant birthrate, *ɛ_i_*(*t*) = *K_i_g,* while temporal heterogeneity was modeled using the seasonal forcing function *ɛ_i_*(*t*) = *K_i_g*(1 sin(2*πt*/365)). In a homogeneous landscape, *K_i_* is the long-term average density per patch, often called the carrying capacity. Throughout, *K* was chosen such that the average number of mosquitoes per human across all patches was 2, i.e., Σ_*i*_ *M_i_*/Σ_*i*_ *H_i_* = 2. Figure 1 was generated using a single patch. Initial conditions were *x* = 0.01 and *Y_i,k_* = *Z_i_* = 0. We generated numerical solutions for 4 y and plotted the last three.

For Figures 2–4, we focused on the relatively simple patterns that form along a spatial transect, a linear array of seventeen patches that can be thought of as a long, rectangular island. We have assumed that *κ_i,j_* = 0 unless two patches are adjacent, and we plot the values at equilibrium. We assume that no humans live in the patches at the extreme ends of the transect, and that all of the mosquitoes leaving one of these edges return to the adjacent patch; thus *κ_1,2_* = *κ_17,16_* = 1, a reflective boundary. Otherwise, we assume that mosquitoes move in either direction at random; thus, *κ_i,j_* = 0.5 for *i* = 2…16 and *j* = *i* ± 1. Mosquito migration was described by the function Φ(H_*i*_) = ζ*e* ^−θH_*i*_^. In Figures 2–4, we used *ξ* = 10 and *θ* = 4. These correspond to a maximum daily flight distance (i.e., without humans) of about ten patches per day.

Adult mosquito emergence for Figures 2 and 4 was *gK*(*P* − 2) in patch 1 (*K* = 2 and *P* = 17); no adults emerged within other patches. The adult emergence rate for Figure 3 was *gK* in each patch with humans (*K* = 2 in patches 2–16).

For Figure 2, human density was 1.0 in patches 2–16. For Figure 3, human density was 0.2 in patches 2–6, 1.8 in patches 7–11, and 1.0 in patches 12–16. For Figure 4 human density was (0,1,2,3,…,15,0)/120. Otherwise, the parameters were the same as in Figure 1.

## Supporting Information

Protocol S1 Additional Methods (285 KB PDF).

## Abbreviations

HBR: human biting rate
EIR: entomological inoculation rate
PIM: proportion of infectious mosquitoes
