# Supplementary material for: The Risk of a Mosquito-Borne Infectionin a Heterogeneous Environment
Source: PLoS Biol. 2004 Oct 26;2(11):e368. doi: 10.1371/journal.pbio.0020368 (PMC524252; doi:10.1371/journal.pbio.0020368)
Supplement: Protocol S1 — (285 KB PDF). [file pbio.0020368.sd001.pdf]

The Risk of a Mosquito-Borne Infection in a Heterogeneous Environment: Supplementary Material

David L. Smith[*], [†], Jonathan Dushoff[‡][†], F. Ellis McKenzie[†][§]

September 20, 2004

Introduction

The following version of the methods is longer and more pedagogical than the published version. The results section includes some additional analysis.

Methods

We use mathematical models to illustrate general principles that may apply to many mosquito-borne diseases, not to make predictions about the distribution of a particular mosquito-borne disease. However, the models we analyze are based on the malaria models of Ross (Ross, 1911). We generate a suite of complex models by elaboration, adding a realistic incubation period, temporal heterogeneity, patchy space and mosquito movement, and spatial heterogeneity (Black & Singer, 1987). By comparing models, we associate an effect with a factor. First, we allow mosquito birth rates to vary temporally, and focus on the temporal changes in the components of EIR (Aron & May, 1982). Next, we illustrate how spatial variability in the distribution of larval habitat generates source-sink relationships in landscapes and leads to variability in the spatial distribution of HBR and PIM. Then, we explore the consequences of heterogeneous human distributions. Host-seeking behavior by mosquitoes can produce mosquito distributions that are more (or less) aggregated than the distribution of humans, generating an uneven distribution in risk. Thus, we develop conceptual models to illustrate which components of the vector biology determine the distribution of risk.

Our intent is to focus on the effects of temporal and spatial heterogeneity. Consequently, we have used a single set of mosquito life-history parameters and a single duration of infection in humans. The parameters are roughly consistent with *Anopheles gambiae* and the infectious period for malaria. Moreover, we allow the distribution or mosquitoes to vary temporally or

[*]Corresponding author: e-mail: smitdave@mail.nih.gov; phone: 301-496-1932
[†]Fogarty International Center, National Institutes of Health, Bethesda, MD 20892
[‡]dushoff@eno.princeton.edu; Ecology and Evolutionary Biology, Princeton University
[§]mckenzel@mail.nih.gov

spatially, and the distribution of humans may vary spatially, but HBR averaged over time or space remains constant. Thus, all of the effects we illustrate are due to temporal fluctuations in mosquito density or to changes in the spatial distribution of humans.

We use a common notation to denote the major components of EIR. We let M denote the population density of mosquitoes, Z the density of infectious mosquitoes, and $z = Z/M$ the proportion of mosquitoes that are infectious. We let a denote the human feeding rate, the number of human bites per mosquito per day. We let H denote the population density of humans.

The transmission dynamics of mosquito-borne diseases are complex, and it is easy to lose sight of what terms such as EIR and HBR actually mean. HBR is the product of the human feeding rate – the number of human blood meals, per mosquito, per day, denoted a – and the number of mosquitoes per human (i.e. $HBR = aM/H$). Therefore, when mosquito density changes, HBR changes proportionally. In contrast EIR is the product of PIM and HBR (i.e. EIR $= z$HBR $= azM/H$). Table 1 lists variable and parameter names and other important terms for the models.

Realistic Incubation Period We first modify the Ross model by incorporating a realistic incubation period into his simple model for the proportion of infected humans and the proportion of infected and infectious mosquitoes. We let x denote the proportion of humans who are infected and infectious. The proportion and density of infected, incubating mosquitoes are denoted y and Y, respectively. We assume that a bite from an infectious mosquito infects a host with probability b, and that each bite on an infectious host infects a mosquito with probability c. We assume that the human infectious period is exponentially distributed with average duration $1/r$.

We assume that the mosquito lifespan is exponentially distributed with a mean lifetime of $1/g$ days. We incorporate a realistic incubation period by subdividing the incubation period into n stages of equal duration; the proportion of mosquitoes that are infected and incubating in stage k is denoted y_k. We assume the incubation period has mean $1/q$ days. For $n = 1$, the probability of surviving the incubation period is $q/(q+g)$, and the duration of the incubation period (for surviving mosquitoes) has an exponential distribution. For $n > 1$, the probability of surviving the incubation period is $(qn/(qn + g))^n$ ($\approx e^{-g/q}$ for large n), and the duration of the incubation period (for surviving mosquitoes) has a Gamma distribution $G(q, n)$, with variance $1/(q^2 n)$. The proportion that become infectious t days after becoming infected is:

$$G(q, n) = \frac{(qn)^n}{(n-1)!} t^{n-1} e^{-tqn} \tag{1}$$

For large n, the incubation period resembles a normal distribution centered at $1/q$; the larger

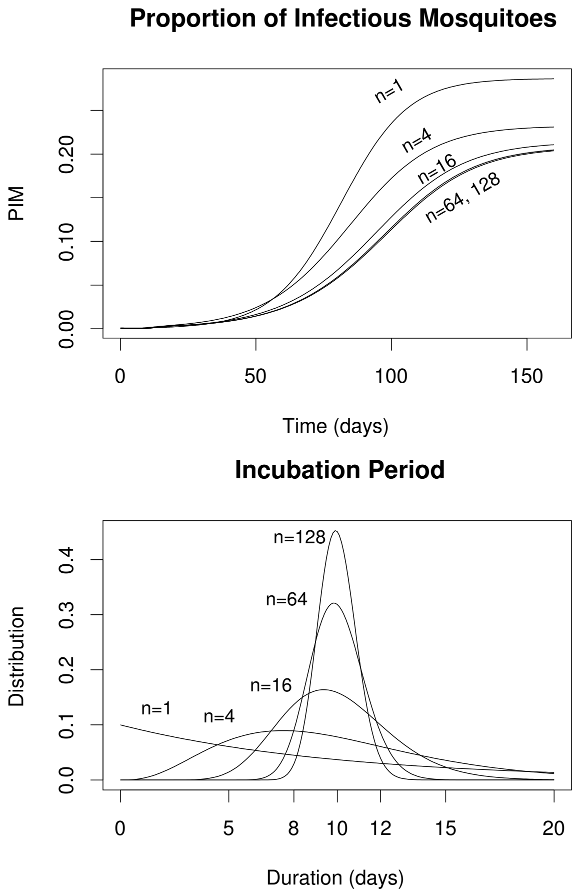

Figure 6: Numerical solutions to Eq.2 for increasingly realistic incubation periods in the mosquito, determined by the number of stages in the incubation period, n. The distribution of the corresponding extrinsic incubation periods are also plotted. For this set of parameters, the dynamics are virtually identical for $n > 64$.

n the smaller the variance (Fig. 6). In the limit as n approaches infinity, the dynamics approach a fixed time delay. We chose n large enough for the incubation period to have a reasonably small variance, and small enough for numerical convenience. Since the distribution for $n = 64$ appears reasonable, and since the dynamics change very little from $n = 64$ to $n = 128$, we have used $n = 64$ for all subsequent figures, unless otherwise specified.

Assuming mosquito population density is constant over time, the proportion of humans and mosquitoes that are infectious changes over time according to the set of $n + 2$ coupled ordinary differential equations:

$$\begin{array}{rcl} \dot{x} & = & ab\frac{M}{H}z(1-x) - rx \\ \dot{y}_1 & = & acx(1 - \sum_k y_k - z) - (qn+g)y_1 \\ \dot{y}_k & = & qny_{k-1} - (qn+g)y_k \\ \dot{z} & = & qny_n - gz \end{array} \tag{2}$$

Numerical solutions to Eqs.2 for various values of n are illustrated in Fig. 6. The entomological parameters were chosen to resemble an efficient vector, such as *Anopheles gambiae* for malaria, ($a = 0.3, b = c = 0.5, 1/g = 1/q = 10$ days). The ratio of mosquitoes per human ($\frac{M}{H}$) was set to 2. The human infectious period for this case is 100 days ($r = 0.01$), roughly consistent with malaria. Initial conditions were $x = 0.01$, $y_k = z = 0$.

Temporal Heterogeneity We extend the model by adding temporal variability in mosquito density. We let $\epsilon(t)$ denote the rate adult female mosquitoes emerge from larval habitat; we do not assume that the emergence of adults is explicitly linked to the density of adult mosquitoes.

Because the mosquito population varies, it is simpler to write the equations in terms of the density of mosquitoes, M, the density of infected and incubating mosquitoes in stage k, Y_k, and the density of infectious mosquitoes Z:

$$\begin{array}{rcl} \dot{x} & = & ab\frac{Z}{H}(1-x) - rx \\ \dot{M} & = & \epsilon(t) - gM \\ \dot{Y}_1 & = & acx(M - \sum_k Y_k - Z) - (qn+g)Y_1 \\ \dot{Y}_k & = & qnY_{k-1} - (qn+g)Y_k \\ \dot{Z} & = & qnY_n - gZ \end{array} \tag{3}$$

Models either employ a constant emergence rate or use the seasonal forcing function $\epsilon(t) = Kg(1 + \sin(2\pi t/365))$; K is the long-term average density, sometimes called the carrying capacity. Fig. 1 was generated using $n = 64$ and $K = 2$; otherwise the parameters are the same as Fig. 6.

Spatial Heterogeneity We further extend the model by subdividing the landscape into a set of patches linked by the movement of mosquitoes. The subscript i is added to variable names to denote the value in the ith patch. Thus, H_i denotes local human population density and x_i the local prevalence of infection in humans. Similarly, M_i is local mosquito population density, and Z_i is the density of infectious mosquitoes. The density of infected mosquitoes in patch i and incubation stage k is $Y_{i,k}$.

Larval habitat and human distributions form a template that determines mosquito distributions and the distribution of risk. The emergence rate of adults in the ith patch is $\epsilon_i(t)$; the emergence of adult female mosquitoes depends predictably on time and location. Following emergence, female mosquitoes spread into surrounding areas seeking blood hosts; they feed, oviposit and then repeat the cycle. We assume that heterogeneity in larval habitat takes the form of differences in quality rather than availability of habitat, *per se*. In other words, we assume that suitable sites for oviposition are distributed throughout the habitat, but that patches may vary in the successful development of adults.

We assume that mosquitoes are more likely to stay in a patch if they encounter a human, and that they are more likely to find humans where humans are more abundant. We let $\phi(H_i)$ denote the per-capita emigration rate of mosquitoes away from patch i regardless of infection status. We assume that $\phi(H)$ is a decreasing function of H; the more humans, the less likely mosquitoes are to leave a patch in search of another blood-meal host. Thus, mosquitoes move more rapidly through patches with low human densities. A parameter, $\kappa_{i,j}$, describes the fraction of mosquitoes leaving patch i that fly to patch j, and $\sum_j \kappa_{i,j} = 1$. Thus, the rate that mosquitoes move from patch i to patch j is $\phi(H_i)\kappa_{i,j}M_i$.

The transmission dynamics are described by the following set of equations:

$$\begin{aligned}
&\dot{x}_i = ab\tfrac{Z_i}{H_i}(1-x_i) - rx_i \\
&\dot{M}_i = \epsilon_i(t) - gM_i - \phi(H_i)M_i + \textstyle\sum_j \kappa_{i,j}\phi(H_j)M_j \\
&\dot{Y}_{i,1} = acx_i(M_i - \textstyle\sum_k Y_{i,k} - Z_i) - (g+qn)Y_{i,1} - \phi(H_i)Y_{i,1} + \textstyle\sum_j \kappa_{i,j}\phi(H_j)Y_{j,1} \\
&\dot{Y}_{i,k} = qnY_{i,k-1} - (g+qn)Y_{i,k} - \phi(H_i)Y_{i,k} + \textstyle\sum_j \kappa_{i,j}\phi(H_j)Y_{j,k} \\
&\dot{Z}_i = qnY_n - gZ_i - \phi(H_i)Z_i + \textstyle\sum_j \kappa_{i,j}\phi(H_j)Z_j
\end{aligned} \tag{4}$$

This patch-based modeling framework is suitable for modeling an array or grid of contiguous habitat or an arbitrary network of patches. We focus on the relatively simple patterns that form along a spatial transect, a linear array of seventeen patches that can be thought of as a long, rectangular island. We have assumed that $\kappa_{i,j} = 0$ unless two patches are adjacent. We assume that no humans live in the patches at the extreme ends of the transect, and that all of the mosquitoes leaving one of these edges return to the adjacent patch; thus $\kappa_{1,2} = \kappa_{17,16} = 1$, a reflective boundary. Otherwise, we assume that mosquitoes move in

either direction at random; thus, $\kappa_{i,j} = 0.5$ for $i = 2 \ldots 16$ and $j = i \pm 1$. Mosquito migration was described by the function $\phi(H_i) = \zeta e^{-\theta H_i}$. In Figs. 2-4, we used $\zeta = 10$ and $\theta = 4$. These correspond to a maximum daily flight distance (i.e. without humans) of about 10 patches per day.

We assume that humans do not move among patches. The density of humans and the productivity of the larval habitat may vary over space. As we change the distribution of humans and larval habitat to explore the effects of spatial heterogeneity, we hold the total emergence rate of adult mosquitoes per human constant; only the distribution of humans and adult emergence changes.

Adult mosquito emergence for Figs. 2 and 4 was $gK(P-2)$ in patch 1 ($K = 2$ and $P = 17$), no adults emerged within other patches. The adult emergence rate for Fig. 3 was gK in each patch with humans $(2-16)$. Adult emergence rate for Fig. 7 was $gK(P-2)\,(1+\sin(2\pi t/365))$ in patch 1; no mosquitoes emerged within other patches.

For Fig. 2, human density was 1 in patches $2-16$. For Fig. 3, human density was 0.2 in patches $2-6$, 1.8 in patches $7-11$, and 1 in patches $12-16$. For Figs. 4 and 7 human density was $(0, 1, 2, 3, \ldots, 15, 0)/120$. Otherwise, the parameters were the same as Fig. 1.

Results

Temporal Heterogeneity Fluctuating mosquito density affects EIR through changes in HBR; transmission increases as mosquito density increases. Following an increase in the rate at which adult mosquitoes emerge, mosquito density and HBR peak (illustrated in Fig. 1). The peak in EIR and the density of infected mosquitoes follows the peak in mosquito density because it takes time for an infectious agent to spread through the human and mosquito populations. Increased HBR leads to secondary increases in the proportion of infected humans, and thus to increases in PIM. As the density of infected mosquitoes declines, decreasing transmission is followed by a decline in the prevalence of infection in humans.

In contrast, larger fluctuations in PIM are generated by the shifting age distribution in fluctuating mosquito populations. Adults emerge uninfected, but they become infected some time after biting infectious humans. Growing populations are dominated by young, uninfected mosquitoes, while shrinking populations are dominated by older mosquitoes. Since the proportion of mosquitoes that are infected and infectious increases with the age of the mosquito, PIM is a proxy for the age distribution of mosquito populations. While populations decline, surviving mosquitoes continue to bite and oviposit but few young mosquitoes emerge, so declining populations have a larger fraction of old mosquitoes. Thus, PIM increases during the dry season as mosquito populations, HBR and EIR decline.

Spatial Heterogeneity The distribution of adults is determined by the distribution of larval habitat, the distribution of blood hosts, and the alternating activities of blood-meal-seeking and oviposition. When mosquito emergence rates and human population distributions are constant over time, the distribution of mosquitoes reaches a static spatial distribution. We focus on the patterns that form along a transect.

In Fig. 2, we assume a single point-source for mosquitoes and a homogeneous distribution of humans. In Fig. 3, the same number of adult mosquitoes emerges, but the spatial distribution of larval emergence is uniform and the distribution of humans varies. We assume that human density is low at one end, high at intermediate distances, and intermediate at the opposite end, approximating a small town with fewer dwellings on the edge nearest a swampy area. In Fig. 4 we combine the two kinds of spatial heterogeneity. Finally, Fig. 7 combines the temporal pattern of of Fig. 1 with the spatial distributions of Fig. 4.

Gradients in EIR away from Larval Habitat When mosquitoes emerge from a point source, the density of mosquitoes tends to decline away from larval habitat, such as a gradient along a transect away from a swamp or river (Fig. 2a). The shape of the gradient is determined by the emergence rate of adult mosquitoes, the mortality of existing mosquitoes, and random drift away from the source. In contrast, PIM increases monotonically away from the source due to a shift in the age-distribution and parity of mosquitoes (Fig. 2a). Young mosquitoes tend to be close to their birthplace because they have moved less; older mosquitoes have moved more and so are dispersed further from the source, on average. The spatial distribution of HBR and EIR reflect the gradients in mosquito density, not the gradient in PIM (Fig. 2a,b). The prevalence of infection in humans declines monotonically (Fig. 2c).

Heterogeneous Distributions of Humans When human populations are distributed heterogeneously, but the larval habitat of mosquitoes is distributed uniformly, adult mosquito distributions become heterogeneous because mosquitoes tend to aggregate around humans. This may or may not lead to an increase in HBR, depending on whether mosquito distributions become more aggregated than the distribution of their human hosts. HBR tends to increase when mosquitoes move rapidly through sparse human populations and stay in areas with dense populations. Thus, mosquito distributions tend to become more aggregated than human distributions when the mosquito species is long-lived with long daily flight distances (see below).

We illustrate this principle for one particular set of parameters that leads to increased mosquito aggregation. The human population is distributed heterogeneously in blocks of low, high, and medium density, approximating a town with a rural population on one side

and an intermediate density population on the other. The distribution of adult mosquitoes is influenced by the distribution of humans (Fig. 3a). Aggregations of mosquitoes form spontaneously at the edges of human settlements simply because mosquitoes tend to move until they find a host. We note that HBR peaks away from town where human population density is lowest and at the edge of town where human population density is highest (Fig. 3b). EIR also peaks at the edge of town, but it is is lowest on the low human density side of town. These movement rules guarantee that the mosquitoes found on the side of town with low human density will tend to be younger, hence PIM is low (Fig. 3a). The prevalence of infection in humans is lowest overall in the patches with low human density (Fig. 3c). This model also makes other surprising predictions–the risk of infection is lowest just outside of the edge of town; the sharp difference in human density at the edge leads to a strong tendency for mosquitoes to be drawn into rather than away from town, decreasing HBR and PIM (Fig. 3b,c).

Heterogeneous Larval Habitat and Human Population When mosquitoes and humans are distributed unevenly, the distribution of mosquitoes and risk may be dominated by either proximity to larval habitats and gradients away from them or by the tendency of mosquitoes to aggregate around humans. The realized pattern depends on the relative distribution of larval habitat and humans, and whether mosquito aggregation around humans increases HBR. We illustrate one kind of pattern for parameters that lead to increased HBR. In this case, human density increases away from larval habitat. The density of mosquitoes peaks a short distance from the source, and the density of infected mosquitoes peaks slightly further away (Fig. 4a). HBR declines monotonically away from the source, but EIR peaks at an intermediate distance (Fig. 4b). The density of infected humans peaks well away from the source, but the fraction of infected humans remains relatively constant near the source, declining abruptly well away from the source (Fig. 4c). Despite the sharp peaks in risk, PIM displays a robust monotonic increase with distance away from the source (Fig. 4a). If the gradient were reversed, so that human density decreases with the distance away from larval habitat, mosquitoes would remain close to the source and mosquito aggregation would be exaggerated, compared with Fig. 2 (not illustrated).

Spatio-Temporal Heterogeneity When the emergence rate of adult mosquitoes varies seasonally (as in Fig. 1), and mosquito larval habitat and humans are distributed heterogeneously (as in Fig. 4), the spatial and temporal patterns are waves focused around mosquito sources. During the wet season, the mosquito population, and hence HBR, expands outward from the source; EIR and the prevalence of human infection follow after brief temporal and spatial lags. As the mosquito population contracts in the dry season, the prevalence of infec-

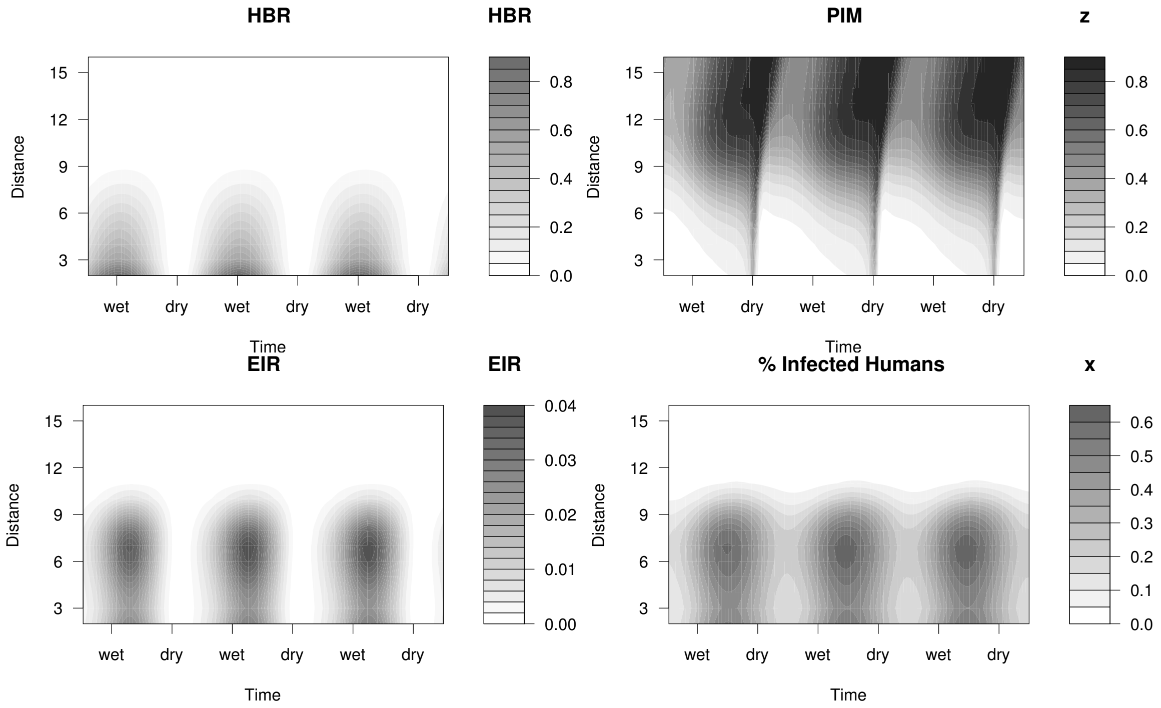

Figure 7: With spatial and temporal heterogeneity, HBR, EIR, and the prevalence of infection in humans tend to expand away from the source in the wet season and contract back toward the source in the dry season. The distribution of humans is heterogeneous, as in Fig. 4, leading to a peak in the risk of infection at a point away from larval habitat. In contrast, PIM peaks far away from the source during the dry season. The ticks on the x-axis mark the peak of the wet or dry season.

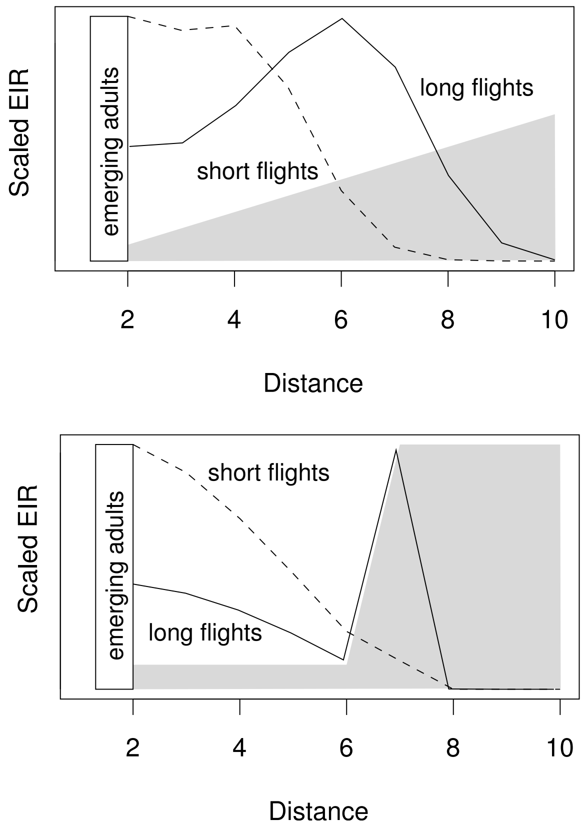

Figure 8: The distribution of risk is affected by the maximum flight distances of mosquitoes. The distribution of EIR reflects the distribution of larval habitat for mosquitoes with short flight distances (dashed), but they come to resemble the distribution of humans for mosquitoes with long flight distances (solid). In order to make comparisons about the distribution of EIR, EIR was scaled; EIR was higher overall when the flight distances were shorter.

tion in humans declines. The prevalence of infection in mosquitoes remains high during the dry season and away from the source, until the population begins to expand again. For this set of parameters, the dry-season reservoir of the infectious agent is the human population. Despite the fact that the prevalence of infection in mosquitoes is high away from the source, mosquitoes remain relatively rare.

Sensitivity Analysis The patterns illustrated in Figs. 2-4,7 are based on a single set of entomological parameters in order to facilitate comparisons in situations in which only the distribution of mosquitoes and hosts vary. The distribution of risk will change for different values of the parameters. We explored the effects of mosquito movement and the duration of incubation period on the distribution of risk (below, and in the supplementary materials).

The tendency of mosquitoes to aggregate at the edges of a town or away from larval

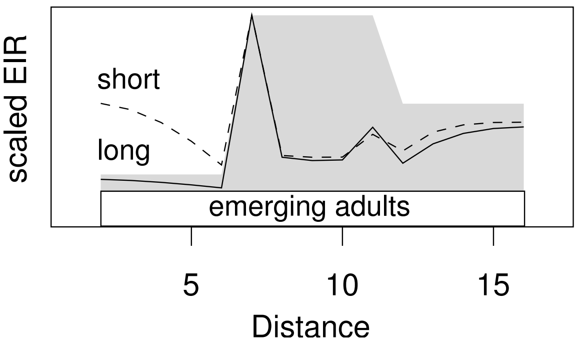

Figure 9: The distribution of risk in heterogeneous environments is affected by the duration of the incubation period. In either case, EIR peaks at the edge of town. For a long incubation, EIR is lower on the side of town where the human population density is lower (solid). The reverse is true for mechanical transmission or a short incubation period (dashed, $q = 10$, $n = 3$). In both cases, HBR outside of town is higher on the low-density side, but when the incubation period is long, mosquitoes migrate away before becoming infectious. In order to make comparisons about the distribution of EIR, EIR was scaled; EIR was higher overall when the incubation period was shorter.

habitat depends on mosquito searching behavior and demography (Fig. 8). Three important parameters that affect these patterns are the maximum daily flight distance of a mosquito, mosquito longevity, and mosquito searching efficiency. The distribution of a mosquito cohort initially reflects the distribution of larval habitat. As mosquitoes search for hosts, the distribution of the cohort shifts to reflect the distribution of human hosts. These tendencies are also reflected in the static spatial distributions of mosquitoes. The distribution of long-lived mosquitoes with long daily flight distances will tend to reflect the underlying distribution of humans, while the distribution of short-lived mosquitoes with short flight distances will tend to reflect the distribution of larval habitat. Mosquito searching efficiency determines the relative rates of movement through habitats that vary in human density. A strong tendency for mosquitoes to aggregate at the edges of dense human populations occurs when mosquitoes move quickly through areas that are sparsely populated by humans and linger in areas that are heavily populated. In other words, mosquitoes tend to become more aggregated than their hosts, increasing HBR, when mosquito searching is relatively inefficient at low human densities.

The distribution of relative risk also changes with the period required for incubation of the infectious agent, with mechanically-transmitted agents at one extreme. All else equal, HBR is higher in areas in which human density is low, since human population density is in the denominator of HBR. On the other hand, HBR may decline in low-human-density areas because mosquitoes tend to move up a gradient of human population density in search of a

blood-meal host. Such migration will tend to lower the average age of mosquitoes in low-human-density patches, especially near the edge of a town (Fig. 9). This will tend to lower PIM for infectious agents with a long incubation period. In contrast, PIM for mechanically transmitted infectious agents will not be as strongly affected, so in comparision, the relative risk may be higher at that same edge of town.

References

Ross, R. 1911. *The prevention of malaria.* John Murray, London.

Black, FL & Singer, B. 1987. Elaboration versus simplification in refining mathematical models of infectious disease. *Ann. Rev. Microbiol.* 41, 677–701.

Aron, JL & May, RM. 1982. The population dynamics of malaria. In *Population Dynamics and Infectious Disease*, (Anderson, RM, ed.), chapter 5, pp. 139–179. Chapman and Hall.

Krantz, SG. 1999. *Handbook of Complex Analysis* p. 118. Boston, MA: Birkhäuser. Formula 9.1.3.

Ruel, JJ & Ayres, MP. 1999. Jensen's inequality predicts effects of environmental variation. *Trends in Ecology and Evolution,* 14, 361–366.
